# Pyrophosphate Stimulates Differentiation, Matrix Gene Expression and Alkaline Phosphatase Activity in Osteoblasts

**DOI:** 10.1371/journal.pone.0163530

**Published:** 2016-10-04

**Authors:** Michael Pujari-Palmer, Shiuli Pujari-Palmer, Xi Lu, Thomas Lind, Håkan Melhus, Thomas Engstrand, Marjam Karlsson-Ott, Hakan Engqvist

**Affiliations:** 1 Division of Applied Material Science, Department of Engineering Sciences, Uppsala University, Uppsala, Sweden; 2 Department of Medical Sciences, Uppsala University, Uppsala, Sweden; 3 Stockholm Craniofacial Centre, Department of Reconstructive Plastic Surgery, Karolinska University Hospital, Stockholm, Sweden; 4 Department of Materials Chemistry, Polymer section, Uppsala University, Uppsala, Sweden; Universite de Lyon, FRANCE

## Abstract

Pyrophosphate is a potent mitogen, capable of stimulating proliferation in multiple cell types, and a critical participant in bone mineralization. Pyrophosphate can also affect the resorption rate and bioactivity of orthopedic ceramics. The present study investigated whether calcium pyrophosphate affected proliferation, differentiation and gene expression in early (MC3T3 pre-osteoblast) and late stage (SAOS-2 osteosarcoma) osteoblasts. Pyrophosphate stimulated peak alkaline phosphatase activity by 50% and 150% at 100μM and 0.1μM in MC3T3, and by 40% in SAOS-2. The expression of differentiation markers collagen 1 (COL1), alkaline phosphatase (ALP), osteopontin (OPN), and osteocalcin (OCN) were increased by an average of 1.5, 2, 2 and 3 fold, by high concentrations of sodium pyrophosphate (100μM) after 7 days of exposure in MC3T3. COX-2 and ANK expression did not differ significantly from controls in either treatment group. Though both high and low concentrations of pyrophosphate stimulate ALP activity, only high concentrations (100μM) stimulated osteogenic gene expression. Pyrophosphate did not affect proliferation in either cell type. The results of this study confirm that chronic exposure to pyrophosphate exerts a physiological effect upon osteoblast differentiation and ALP activity, specifically by stimulating osteoblast differentiation markers and extracellular matrix gene expression.

## Introduction

Pyrophosphate sequesters calcium in the body, thereby allowing supersaturated levels of calcium to exist in the blood, while also actively preventing pathological calcification/mineralization [1, 2]. This biochemical activity is reversed in osseous tissue, where alkaline phosphatase cleaves pyrophosphate to produce inorganic phosphate and free calcium[3]. Calcium pyrophosphate is also a potent mitogen that mimics the effect of serum and growth factors, and can push fibroblasts that have entered a quiescent state back into proliferation[4–6]. Though alkaline phosphate is a critical participant in osseous mineralization, few studies have investigated the physiological effects of exogenous pyrophosphate on osteogenic cells[7–9].

The molecular basis of pyrophosphate stimulated proliferation is poorly understood. However, basic calcium phosphate crystals (BCP) can stimulate similar effects, such as proliferation in osteoblasts, and the mechanism underlying these changes are well understood. BCP include hydroxyapatite, octacalcium phosphate and brushite crystals[10–13]. Hydroxyapatite BCPs are stable, acting as a calcium reservoir that release calcium under acidic conditions. Similarly, pyrophosphate can release calcium under acidic conditions, like those found in the endosome, and enzymatic (alkaline phosphatase) environments, such as matrix vesicles at the site of mineralization [10, 14–16]. Despite the similarities between BCP and pyrophosphate, it is not known whether exogenous pyrophosphate is taken up by cells, whether gene expression changes occur similar to BCP, i.e., rapid upregulation of cyclo-oxygenase-2 (COX-2) at 4 and 32 hours, or whether pyrophosphate stimulates a mitogenic response in osteoblasts[10]. Interestingly, there are multiple forms of pyrophosphate including soluble sodium pyrophosphate, amorphous calcium pyrophosphate (ACPPi), and calcium pyrophosphate dihydrates (monoclinic crystal mCPPD, triclinic crystal tCPPD, etc.). The physiochemical properties, such as precipitation and dissolution rates, may differ based upon the pyrophosphate form[17].

In osseous tissue culture models pyrophosphate is reported to inhibit mineralization by binding directly to mineralization active site/faces and preventing further crystal growth, by inhibiting the production of free phosphate from organic molecules such as beta-glycerol phosphate, and by increasing expression of inhibitory proteins[8, 18]. Paradoxically, pyrophosphate can also stimulate differentiation and mineralization by upregulating ALP expression and enzymatic activity, thereby increasing the local concentration of calcium and phosphate upon cleavage by alkaline phosphate, and by stimulating MAP kinases and PGE2[19–21]. Ex vivo studies have demonstrated that the calcium bound by exogenous pyrophosphate localizes to the site of active mineralization (i.e. matrix vesicles), further supporting a facilitative physiological role for exogenous pyrophosphate[16].

Pyrophosphate also participates in the osseous integration of orthopedic biomaterials. Both commercial and research grade bioceramics often contain pyrophosphate, where it is used as a crystal growth inhibitor to control the mechanical properties and setting time of ceramics[22]. Scaffolds and ceramics that incorporate pyrophosphate as an active component have reported enhanced bioactivity and mineralization[23–26]. Clinical findings in humans have also confirmed that bioceramics containing pyrophosphate integrate better, with optimal resorption rates and greater mineralization in vivo[23, 27–29]. In the present study we investigated whether exogenous pyrophosphate a) can act as a mitogen in osteoblasts similar to prior studies in fibroblasts, or BCPs in osteoblasts, b) the concentration range over which pyrophosphate affects differentiation and ALP activity, c) can alter osteogenic gene expression in differentiating pre-osteoblasts, and d) whether the biological mechanism underlying the effects of CPPi is similar to what has been reported for other calcium phosphate crystals, CPPD or BCPs.

## Methods

### Materials

All materials, including sodium pyrophosphate dibasic (>99%), L-ascorbic acid (>99%), and beta-glycerol phosphate disodium (>99%) were purchased from Sigma-Aldrich,(Sigma, Steinheim, Germany) unless otherwise indicated.

### Material characterization

A 50mM sodium pyrophosphate stock solution was created by dissolving sodium pyrophosphate in deionized water, neutralized with 52.1μM NaOH, followed by filtration (0.2 μm PES). This stock solution was made fresh immediately prior to treatment. Calcium pyrophosphate precipitant was obtained by diluting sodium pyrophosphate to a final concentration of 1mM or 100μM in alphaMEM (Gibco), at 1mL/cm^2^, 37°C in a humidified atmosphere of 5% CO2 for 48 hours. Sodium pyrophosphate formed an insoluble calcium salt at 100μM in alpha-MEM, and between 125–200μM in DMEM media. Crystals were isolated by centrifugation at 3,000g for 1 hour, and air-dried for 48 hours. The x-ray diffraction (XRD) spectra of sodium pyrophosphate precipitant was obtained with a Bruker D8 advanced XRD machine. FTIR spectra were obtained on a Bruker Tensor 27 FTIR machine using a Platinum ATR attachment. SEM images were obtained on a Leo 1550 SEM (Zeiss).

### Cell Culture

MC3T3-E1 murine calavarial osteoblasts (subclone 14) cells and human osteosarcoma SAOS-2 cells were purchased from American type tissue culture (ATCC). MC3T3 and SAOS-2 were subcultured in alpha-MEM (Gibco) and DMEM (Gibco), respectively, with 10% fetal bovine serum (FBS) and 1% penicillin/streptomycin at 37°C in a humidified atmosphere of 5% CO2, with complete media replacement every 48 hours. Osteogenic media included 50ug/mL of ascorbic acid and 10mM beta glycerol phosphate[30].

### Proliferation

At predetermined intervals following treatment (24 hours for MC3T3 and 36 hours for SAOS-2) the media was replaced with 150uL (96 well) of fresh media containing 10% alamar blue. After 1 hour incubation at 37°C, 100uL of the reduced solution was transferred to a black well plate, and the fluorescence was detected at (570nm excitation, 590 emission) on a Tekan microplate reader. The background was subtracted from each sample and the average values were normalized to the PBS treated controls and expressed as percent survival.

### Treatment

MC3T3 cultures were treated with 10-fold concentrated stock solutions that were diluted directly into the medium. The composition of, and mitogenic response to, precipitated pyrophosphate salts may differ based on the amount of calcium and phosphate in the media[6]. To closely mimic MC3T3 treatment conditions, SAOS-2 were treated with calcium pyrophosphate crystals that had been freshly precipitated in serum free alpha MEM media for 48 hours (1mM sodium pyrophosphate). Throughout this publication the term *pyrophosphate* refers to the precipitant of sodium pyrophosphate in cell culture media (calcium pyrophosphate). Crystals were isolated by centrifugation at 3,000g for 1 hour, followed by resuspension in DMEM, in 1/10^th^ the original media volume (10x concentration), and direct dilution into culture medium. In a separate treatment regimen MC3T3 media was replaced with low serum (0.5% FBS, serum starvation) media for 16 hours prior to, and during, treatment with sodium pyrophosphate to control for cell cycle asynchronicity.

### Alkaline phosphatase activity

Differentiation samples were seeded at 10,000 or 13,000 cells/cm^2^, for MC3T3 and SAOS-2 respectively, and allowed to reach confluence for 48 hours. After 3, 5, and 7 days in SAOS-2, or 5, 7, and 10 days in MC3T3, the plates were gently rinsed in warm PBS, 200uL of lysis buffer (20mM Tris, 1mM MgCl, 0.1mM ZnCl, 0.1% Triton-X 100) was added and samples were stored frozen at -20°C for later analysis. The lysates were freeze/thawed three times and a 25uL aliquot was taken from each well and combined with 50uL of alkaline phosphatase substrate for 10 minutes. The reaction was stopped by adding 25uL of 3M NaOH, and the conversion of substrate p-nitrophenylphosphate into free 4-nitrophenol was determined by spectrophotometer (Tekan plate reader) at 405nm. A 50uL aliquot was taken from each well and combined with an equal volume of micro-BCA working solution (Sigma, Steinheim, Germany), per the manufacturer’s instructions, incubated at 37°C for 1 hour, and the absorbance was read at 562nm on a Tekan plate reader. ALP and BCA readings were compared to a standard curve of 4NP and BSA respectively, and the calculated alkaline phosphatase activity was normalized to the protein content for each well. Experiments were replicated twice, with 4 samples per group in each experiment. The data was plotted after normalizing the ALP activity/ug/min to the control, for each experiment.

### RNA Quantitation

MC3T3 cells were seeded as per ALP activity studies and allowed to reach confluence. Every 48 hours the media was replaced with osteogenic media and sodium pyrophosphate. After 4 hours, 1, 1.5, 3, 5, 7, or 10 days the media was removed, rinsed with PBS, Tri-reagent (Sigma, Steinheim, Germany) was added (1mL /10 cm^2^), and samples were stored frozen at -20°C. Total RNA was isolated according to the manufacturer’s protocol, and quantified with a nanodrop 1000 (Thermo scientific) machine. cDNA was synthesized with 0.5 ug of RNA, using a high capacity cDNA reverse transcription kit (Applied Biosystems) according to the manufacturer’s instructions. The following genes were selected for investigation: osterix is a transcription factor, activated downstream of master regulator RUNX2, that is required for osteoblast mineralization[31]; collagen 1 (COL1) is an extracellular matrix protein that stimulates differentiation of MC3T3 osteoblasts[32]; alkaline phosphatase (ALP), the most commonly used indicator of osteogenic differentiation, regulates the local concentration of calcium, phosphate, and mineralization in vivo via cleavage of organic and inorganic phosphates such as pyrophosphate or beta-glycerol phosphate; osteopontin (OPN) is a matrix protein that negatively regulates crystal growth (mineralization) based upon it’s phosphorylation state[8]; cyclo-oxygenase 2 (COX-2) encodes the enzyme that produces PGE-2, a strong bone anabolic stimulator, and is conditionally expressed in response to stress; osteocalcin (OCN) is a matrix protein that is an indicator of mature differentiated osteoblasts[33]. Pyrophosphate is normally produced intracellularly as part of normal nucleic acid metabolism, and is transported out of the cell by the gene product of ankylosis protein homolog (ANK)[3]. The primers for beta-actin (Mm02619580_g1), osteopontin (Mm00436767), alkaline phosphatase (Mm01187117_m1), osterix (Mm00504574_m1), collagen1 (Mm00801666_g1), cyclo-oxygenase 2 (Mm03294838_g1), ANK (Mm00618325_m1), osteocalcin (Mm03413826_mH). were purchased from Applied Biosystems and used as suggested by the manufacturer. Quantitative PCR was performed using an CFX96 Touch™ Real-Time PCR detection system (BioRad) and Rotorgene RG-3000 machine (Corbett research) and analyzed with the manufacturer supplied software (AB software, Rotorgene version 6.1). The reaction conditions were: 50°C for 2 minutes, 95°C for 10 minutes, followed by 40 cycles of: 95°C for 15 seconds and 60°C for 1 minute. The relative quantification method was used to normalize each gene to a housekeeping gene (beta-actin).

### Statistics

Each data point represents an average of 3 (qPCR), 4 (ALP activity) or 5 (proliferation) samples, with 2 replicates of each experiment. Statistical significance was determined by ANOVA, with Tukey HSD post-hoc analysis for qPCR experiments. All groups were normally distributed (Shapiro-Wilk p>0.05), with homogenous variance (Levine's p>0.05), except for Collagen-1 expression on day 7. The data for collagen-1 were pooled from both experiments and analysed with non-parametric Kruskal Wallis. While both tests reached significance (p = 0.006 ANOVA, p = 0.034 Kruskal Wallis), the latter, non-parametric test value has been reported for collagen-1. A subsequent mega analysis (ANCOVA) was used to confirm the difference in the means between treated and control groups, after pooling all sample data using “experiment replicate number” as the covariate. An ANVOA with Dunnet’s-T test was used for multiple dose comparisons to control (proliferation and ALP activity).

## Results

**Fig 1:**
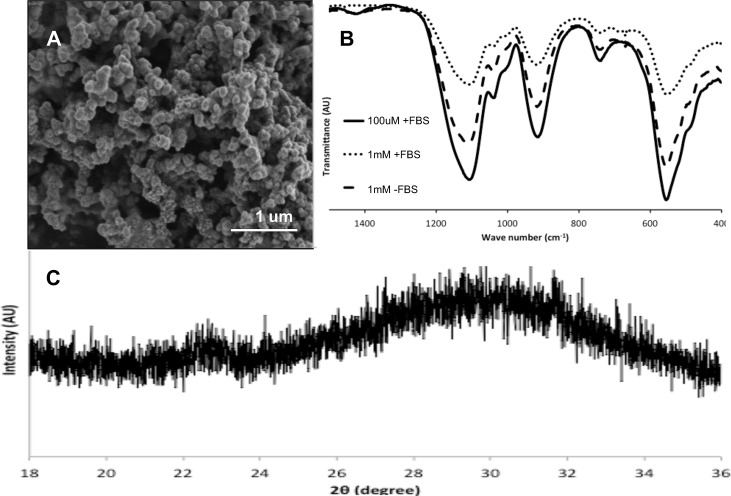
Characterization of calcium pyrophosphate precipitant. The SEM morphology (A) of the precipitant is similar to that of other amorphous calcium phosphates (nanometer diameter spheres). FTIR (B) and XRD (C) analysis confirm an amorphous composition.

Sodium pyrophosphate rapidly forms an insoluble precipitant within 15 minutes, at 100μM in alpha-MEM or approximately 150μM in DMEM media. Increasing concentrations formed a greater amount of precipitant, with an approximate yield of 0.5 mg/mL at 1mM and 0.01 mg/mL at 100μM in alpha MEM. SEM images of the dried precipitant (Fig 1A) reveal a spherical/ amorphous morphology, ranging from 50 to 100nm in diameter, similar to what has been reported by Gras and Leeuwin et al. [17, 34]. The XRD spectra (Fig 1B) confirms an amorphous composition, with a broad peak at 30°. FTIR analysis of the precipitant (Fig 1C) confirmed an amorphous phase as the spectra was an exact match to the results reported in Gras et al. for synthetic amorphous calcium pyrophosphate[17]. The peak and shoulders at 1128, 1058 and 1014 cm^-1^ correspond to the Ca-PO4 bond, while the 925 and 565 cm^-1^ peaks correspond to the P-O-P and P-O_4_ bonds, respectively [35, 36]. The presence or absence of fetal bovine serum and penicillin/streptomycin, common substances in tissue culture that may interfere with or be incorporated into the precipitant, did not affect the precipitant as the FTIR spectra are identical under both conditions. It has been reported that different precipitants are obtained depending on the concentration of sodium pyrophosphate, calcium and phosphate in the precipitation solution. However, the FTIR spectra appear identical for precipitants at 1mM and 100uM sodium pyrophosphate.

**Fig 2:**
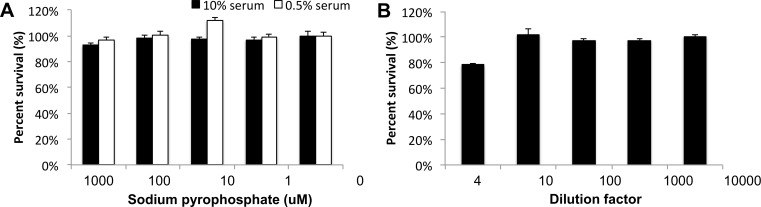
**Proliferation of MC3T3 (A) and SAOS-2 (B) after exposure to pyrophosphate.** The effect of pyrophosphate during serum starvation are indicated in (A) by the 0.5% serum treatment group.

Pyrophosphate did not affect proliferation in MC3T3 or SAOS-2 over 10-fold ranges between 1uM and 1mM (Fig 2). When osteoblasts were exposed to pyrophosphate under serum starvation conditions, to control for variation due to cell cycle phase, no difference in proliferation was observed (Fig 2A).

**Fig 3:**
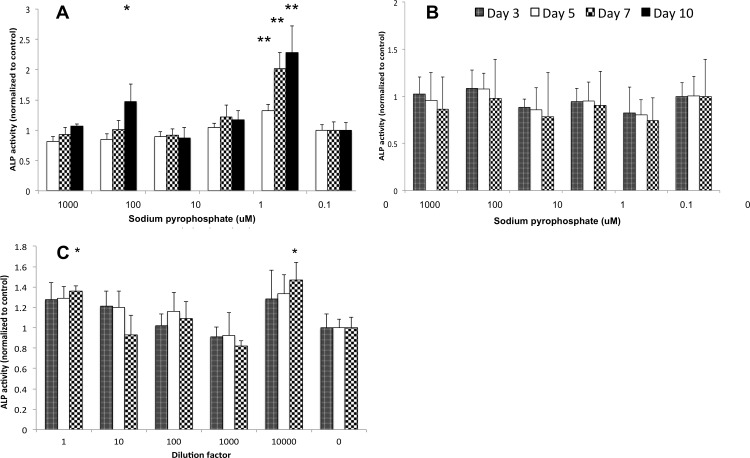
ALP activity after treatment with pyrophosphate. ALP activity in MC3T3 (A) was maximally stimulated by 100uM and 0.1uM sodium pyrophosphate. In SAOS-2 sodium pyrophosphate failed to stimulate ALP activity (B). When the amorphous calcium pyrophosphate precipitant was isolated and diluted from alha MEM media, into SAOS-2 cells in DMEM media (C), ALP activity was stimulated at 1-fold and 10,000-fold dilutions. Differences between treatment and untreated group means that were statistically significant are indicated with * (p < 0.05) or ** (p < 0.01). Each data point represents the ALP activity normalized to protein content at 3, 5, 7 or 10 days of continual exposure to pyrophosphate.

Early stage osteoblasts responded to sodium pyrophosphate with increased ALP activity in a bimodal response (Fig 3A). Peak ALP activity in MC3T3 reached 150% and 250% of untreated controls after exposure to 100μM (P = .048, .005) or 0.1μM (P = < .0001, and < .0001) sodium pyrophosphate, respectively (Fig 3A). Sodium pyrophosphate does not precipitate into calcium pyrophosphate as readily in SAOS-2 medium (DMEM). In addition, higher variance in ALP activity was observed in SAOS-2 than in MC3T3. To control for the variable precipitation rate SAOS-2 cells were treated with either sodium pyrophosphate (Fig 3B), or precipitant that was isolated from sodium pyrophosphate treated alpha-MEM (Fig 3C). Sodium pyrophosphate concentrations of 100μM stimulated peak ALP activity by 20–30%, though this difference was not statistically significant (Fig 3B). When calcium pyrophosphate precipitant was collected directly from MC3T3 medium and diluted 1, 10 or 10,000-fold into SAOS-2 cells (Fig 3C) peak ALP activity was increased 30% and 50% compared to untreated controls (P = <0.001, 10,000-fold).

**Fig 4:**
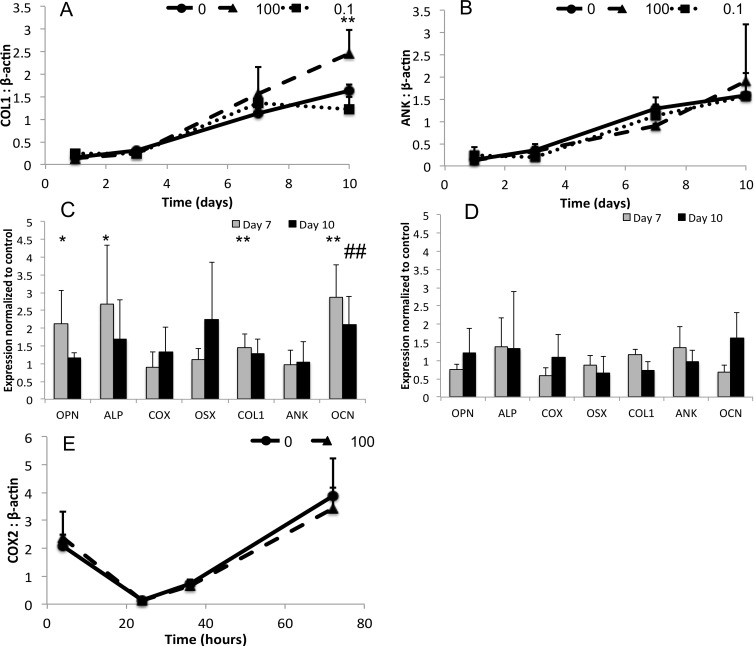
Gene expression in MC3T3 after continual exposure to pyrophosphate. Expression of representative genes OCN (A), and OSX (B), and peak expression of all genes in the 0.1uM (C) or 100uM (D) treatment groups, expressed as fold increase over control group. COX-2 expression during the first 36 hours is shown in (E). Statistically significant differences are indicated with *, # (p < 0.05) or **,## (p < 0.01) for comparison with untreated controls at the 7 day (*) or 10 day (#) time point.

Within the first three days of exposure to pyrophosphate there was no significant expression differences for any gene, when comparing treatment and control groups. Representative time course expression profiles of genes that were affected (COL1) and unaffected (ANK) by pyrophosphate are shown in Fig 4A and 4B, respectively. ANK, OSX and COX expression was not significantly affected by pyrophosphate exposure throughout the 10 day period (Fig 4B–4D). In contrast, extracellular matrix protein OPN, COL1 and OCN expression was elevated 1.5–2.5-fold, 1.5-fold and 3-fold respectively, (P = 0.014; 0.034; <0.001) after 7 days of exposure to 100μM sodium pyrophosphate (Fig 4A–4D). Alkaline phosphatase expression increased 1.5–3 fold over control for 100μM (P = 0.016) and 0.1μM treatment groups, though this difference failed to reach statistical significance for the 0.1μM treatment group. In a similar model of mitogenic calcium phosphate precipitant, basic calcium phosphate crystals (BCP), COX-2 expression is elevated after 4 and 36 hours of exposure, therefore these additional time points were included in this study[10]. When COX-2 expression was examined during the first 72 hours of exposure there was no significant difference between the 100μM treatment group and control (Fig 4E).

## Discussion

Pyrophosphate is a diphosphate ester that prevents pathological mineralization by chelating free calcium [1, 37]. In the present study spherical, amorphous calcium pyrophosphate (ACPPi) precipitated in alpha-MEM media and stimulated ALP activity in MC3T3 and SAOS-2 cells. Pyrophosphate can mimic the effects of serum in fibroblasts, however we did not observe an effect on osteoblast cell proliferation under normal serum or starvation conditions. Alkaline phosphatase activity increased in response to pyrophosphate, biphasically with optimal stimulation at 100μM and 0.1μM sodium pyrophosphate, in agreement with the results of Addison et al [8].

While numerous studies have reported improved osseous bioactivity on bioceramics containing pyrophosphate, often the exact form of pyrophosphate (CPPD crystals, ACPPi, sodium pyrophosphate) or combination thereof, varies between, and even within, each study [23–26]. For example, Grover et al demonstrated enhanced mineralization on ceramics containing amorphous calcium pyrophosphate in vivo, though CPPD crystals, rather than ACPPi, were subsequently used to determine whether the bioactivity was due to enzymatic production of phosphates or Chatelier's principle[23]. In the present study calcium pyrophosphate stimulated osteogenic differentiation and expression of matrix genes in early stage osteoblasts, similar in magnitude to what has been reported for osteogenic biomaterials [38–40]. A standard osteogenic gene expression threshold to determine whether a material is definitively osteogenic has not been proposed. However, orthopedic biomaterials including PLGA, hydroxyapatite and collagen gels are often reported to be “osteogenic” when the expression of OCN, OPN, ALP or COL1 exceeds 1.5–2 fold of control values[38–40]. Osteogenic genes ALP, COL1, OPN and OCN increased in pre-osteoblasts after exposure to pyrophosphate, though given the variability we observed in OSX expression, we cannot conclusively rule out the involvement of OSX. Similarly, an increase in intracellular pyrophosphate has been reported to increase collagen expression, without affecting canonical regulator genes such as ATF4[41]. Kim et al. reported a 1.5-fold increase in both COL1 and osterix after exposure to 500uM pyrophosphate, though they observed no change in ALP expression at 12 days[9]. Addison et al observed an increase in ANK, ALP and OPN expression following treatment with 2–10μM pyrophosphate for 12 days[8]. In our hands lower concentrations of pyrophosphate (0.1 μM) stimulated ALP activity but did not affect osteogenic gene expression. The reason that our results differ slightly from Addison are not obvious, though we have examined earlier time points (1–10 days versus 12 days) and different concentrations. It should be noted that Kim et al treated MC3T3 with pyrophosphate during the first 6 of 12 days during differentiation, while Addison et al treated MC3T3 only during the later 6 of 12-day differentiation cultures. While these studies report osteogenic gene expression changes in agreement with the present results, both studies have focused on the effects of short-term exposure (6 days). In the present study pre-osteoblasts were continually exposed to pyrophosphate to mimic the continual release of pyrophosphate from an implanted bioceramic. The 100μM treatment was selected because it is the point at which calcium pyrophosphate precipitates out of solution, and because it stimulates proliferation in other cell types. While an increase in OPN and ALP expression may reflect a positive feedback response to the inhibitory effects of pyrophosphate on mineralization, COL1 and OCN expression reflect longer-term extracellular matrix changes that support enhanced osteogenic differentiation. Collagen 1 is a necessary initiator of differentiation in MC3T3 osteoblasts, and a required substrate for mineralization to occur. Osteocalcin is expressed only in mature osteoblasts, and OCN overexpression is capable of causing osteochondrogenic differentiation of, and mineralization by, vascular cells [42–44].

Pyrophosphate is concentrated into matrix vesicles by the membrane transporter ANK [45]. While no transporter or transport mechanism has been described that can transport extracellular pyrophosphate into the cell, chemically labeled organic pyrophosphates have been observed accumulating inside the cell[46]. In contrast, basic calcium phosphate crystals are known to enter the cell by endocytosis and stimulate gene expression changes by releasing calcium under the increasingly acidic conditions of the endosome/lysosome [4, 10, 12, 15]. We hypothesized that pyrophosphate might act via a similar mechanism, as pyrophosphate can also release calcium under acidic conditions. Though CPPD has been reported to stimulate PGE-2 production, osteoblasts exposed to calcium pyrophosphate did not respond with rapid COX-2 gene expression, or proliferation, as has been reported with BCPs [10, 21]. This is not entirely surprising, given that the proliferative effects of pyrophosphate have only been reported under low serum and quiescent conditions [5, 6]. The present data does not support the involvement of endocytosis, or the intracellular release of calcium within endocytic vesicles as seen with BCPs, in response to pyrophosphate exposure.

The exact mechanisms underlying the gene expression and protein activity changes in response to extracellular pyrophosphate exposure are not well understood. In the present study the expression of pyrophosphate transporter, ANK, was not affected by pyrophosphate, suggesting that ANK does not participate in the osteogenic response to extracellular pyrophosphate. The products of pyrophosphate cleavage, calcium and inorganic phosphate, can stimulate gene expression changes directly. Elevated phosphate can stimulate COL1, OPN and ALP[47, 48]. Both excessive calcium and phosphate can activate Erk1/2 [9, 47–49]. However, phosphate uptake blocker and pyrophosphate analog, foscarnet, does not inhibit the effects of exogenous pyrophosphate, suggesting that breakdown of PPi into inorganic phosphate may not explain observed gene expression changes[8, 50].

The inhibitory effects of calcium pyrophosphate on mineralization, in vitro, appear to contradict the reports by Grover et al and others of enhanced mineralization on ceramics containing pyrophosphate, in vitro, and in vivo. It is possible that the form (anchored as a substrate versus a freely dispersed precipitant, amorphous calcium pyrophosphate versus CPPD, nano-sized precipitant versus micrometer sized crystals within a bioceramic surface) and location (apically located precipitant versus basally located within a biomaterial surface for cell adhesion, where matrix vesicles and basal body structures containing ANK reside) of pyrophosphate can affect how cells respond. The physical properties of calcium pyrophosphate, such as solubility, particle size, uptake or hydrolysis rate are also important variables that can vary depending on the synthesis, purification, sterilization and storage conditions [4, 15, 21]. The sterilization process of autoclaving and even the commonly used dispersion process of ultrasonication, can alter the crystal structure of amorphous calcium pyrophosphate towards monoclinic and triclinic-like crystals [17, 34]. It is, therefore, important to characterize the exact form of pyrophosphate used in cellular testing. Collectively, we have shown that pyrophosphate stimulates osteogenic gene expression, and phosphatase activity in early and late stage osteoblasts. Future studies should compare the effects of freely precipitated pyrophosphate and substrate/surface bound pyrophosphate on mineralization, whether the particle size and crystalline forms of pyrophosphate affect osteogenic cells identically, and investigate the effects of pyrophosphate on osteoclasts and osteocytes.

## Conclusion

The present study sought to characterize the effects of chronic exposure to pyrophosphate, and to extend the knowledge provided by Addison and Kim et al. We have demonstrated that chronic pyrophosphate exposure stimulates genes involved in osteoblast differentiation, specifically in matrix secretion, and ALP activity. We also report that the effects of pyrophosphate on osteogenic gene expression are dissimilar to effects of basic calcium phosphate crystals.
